# Analysis of Non-communicable disease prevention policies in five Sub-Saharan African countries: Study protocol

**DOI:** 10.1186/s13690-016-0137-9

**Published:** 2016-06-22

**Authors:** Pamela A. Juma, Shukri F. Mohamed, Jennifer Wisdom, Catherine Kyobutungi, Samuel Oti

**Affiliations:** African Population and Health Research Center (APHRC), Manga close off Kirawa road, Kitisuru, P.O. Box, 10787-00100, Nairobi, Kenya; Wisdom Consulting, 416 Florida Ave NW, Box 26382, Washington DC, 20001 USA

**Keywords:** Multi-sectoral approach, Best-buys, Policies, non-communicable diseases, Sub-Saharan Africa

## Abstract

**Background:**

The burden of non-communicable diseases (NCDs) and their risk factors is increasing in sub-Saharan Africa, and there have been calls for adopting a multi-sectoral approach in developing policies and programs to address this burden. Evidence exists largely from high-income countries on the success (and lack thereof) of multi-sectoral approach in improving population level health outcomes. In sub-Saharan Africa, there is limited research on the application and success of multi-sectoral approach in the formulation and implementation of policies aimed at prevention of non-communicable diseases. Therefore, this protocol describes a study that aims to primarily generate evidence on the extent to which multi-sectoral approach has been applied in developing policies to prevent non-communicable disease in six countries in sub-Saharan Africa –Kenya, Malawi, Nigeria, Cameroon, Togo and South Africa.

**Methods/Design:**

The study applies a multiple case study design. Data will be collated mainly through document reviews and key informant interviews with the relevant decision makers in various sectors. In each country, a detailed case study analysis will be undertaken of any policy/policies developed, adopted and implemented, aimed at implementing the World Health Organization recommended “best buys” for non-communicable disease prevention. These case studies will be conducted by research teams in each country; each team includes a senior research fellow supported by a doctoral student, and research assistants.

**Discussion:**

Uptake of the evidence generated from the case studies will be ensured by systematic engagement with policy makers in each country throughout the research process. Ultimately, a forum of experts will be convened to generate actionable recommendations on the use of multi-sectoral approach in non-communicable disease prevention policies in the region.

## Background

The burden of non-communicable diseases (NCDs) and their risk factors are increasing in sub-Saharan Africa [1–3]. NCDs account for 63 % of mortality globally and the 80 % of NCD-related deaths that occur in low and middle income countries [4, 5]. Current projections indicate that by 2020, the largest increase in NCD-related deaths will occur in Africa and by 2030, NCD-related deaths will exceed (by 75 %) the combined deaths from communicable diseases, nutritional, maternal and neo-natal deaths [6]. Out of the many NCDs, four – cardiovascular diseases, diabetes, cancers and chronic respiratory illnesses – have been identified as being responsible for the majority (75 %) of all NCD-related mortality [3]. These four NCDs also share a set of four risk factors: tobacco use, unhealthy diet, harmful alcohol use and physical inactivity. These factors have been linked to increase in preventable morbidity and disability in the region, and globally currently causing over 36 million deaths annually. More than 9 million (a quarter) of these deaths occur before the age of 60 years [6–8].

To address the burden of NCDs and their associated risk factors, the World Health Organization (WHO) has advocated for a ‘whole-of-government’ approach in its most recent global strategy for NCD prevention and control [9]. A whole-of-government approach denotes public service agencies working across portfolio boundaries to achieve a shared goal and an integrated government response to particular issues. Furthermore, the global NCD strategy recommends that such the whole-of-government approach should be aimed at addressing the social determinants of health. The social determinants of health are those conditions in which people are born, grow, work, live, and age, and the wider set of forces and systems shaping the conditions of daily life [10]. These forces and systems include economic policies and systems, development agendas, social norms, social policies and political systems. Social determinants of health play a great role in creating the environment that influences population risk and individual behavior. These determinants lie beyond the purview of the health sector and several non-health sectors (e.g., agriculture, education, urban planning, and transportation) play a big role in shaping the NCD environment. A key component of the whole-of-government approach is the multi-sectoral approach (MSA). MSA in the context of health refers to actions undertaken by sectors outside the health sector, possibly, but not necessarily, in collaboration with the health sector, on health or health- related outcomes or the determinants of health or health equity [11]. As such, action within and between sectors, at the local, regional, provincial, national, and global levels, is needed to influence the social and economic landscape that enables the health and well-being of the population. Thus successful NCD prevention therefore needs MSA to address factors that impact on the physical, social and political environment that shapes individual choices.

The first Global Ministerial Conference on Healthy Lifestyles and Non Communicable Disease Control held in Moscow in April 2011 set the stage for the high level UN-Summit on NCD in New York and the subsequent political declaration in September 2011 [6]. The Moscow Declaration on NCDs that emanated from the ministerial conference contained a commitment from governments to develop multi-sectoral public policies that create equitable health promoting environments that enable individuals, families and communities to make healthy choices, lead healthy lives as they give priority to NCD prevention and control, ensuring complementarity with other health objectives and to strengthen the engagement of other sectors [8]. With this regard, governments pledged to promote, establish or strengthen, and implement by 2013, multi-sectoral national policies and plans for the prevention and control of NCDs, taking into account the WHO 2008–2013 Action Plan for the Prevention and Control of NCDs. This plan identifies MSA as a cornerstone for NCD prevention at population level. It also identifies several “best buys” for NCD prevention including measures to reduce the four common risk factors of tobacco use, unhealthy diet, physical inactivity and the harmful use of alcohol that would deliver the greatest benefit in reducing population level risks in a cost-effective manner [12]. Table 1 provides examples of the NCD prevention “best buys.” It is evident that the implementation of these “best buys” requires MSA. For example, raising taxes and enforcing advertising bans/restrictions on tobacco and alcohol will require the involvement of ministries of finance, health, information, as well as the legislative arms of government, law enforcement, and the media.

**Table 1:** WHO recommended “best buys” for NCD prevention and control

Risk factor	“best buy interventions”
Tobacco use	Raise taxes on tobacco
	Protect people from tobacco smoke
	Warn about the dangers of tobacco
	Enforce bans on tobacco advertising
Harmful use of alcohol	Raise taxes on alcohol
	Restrict access to retailed alcohol
	Enforce bans on alcohol advertising
Unhealthy diet and physical inactivity	Reduce salt intake in food
	Replace trans-fat with polyunsaturated fat
	Promote public awareness about diet and physical activity (via mass media)

Evidence exists on successful MSA in tobacco control initiatives in several settings [11, 13]. However, there is limited research on the application and success of MSA for the control of other risk factors (i.e., harmful use of alcohol, physical inactivity and unhealthy diets) in sub-Saharan Africa. Evidence also exists on the use of MSA in improving population level health outcomes. A systematic review of multi-sectoral interventions to address social determinants of health while improving health equity was recently conducted [13]. In general, the review found that there were successful examples of MSA that resulted in health impacts, but it had substantial limitations in that most interventions did not specifically address the social determinants of health, most only included developed countries, they focused on interventions in specific settings, such as schools, they provided little detail about process, and they often did not articulate how relationships between sectors contributed to outcomes.

This paper describes, “Analysis for Non-communicable Disease Prevention Policies in Africa (ANPPA),” a research project that aims to fill the gaps highlighted by the systematic review above. The main contribution of this research will be the generation of evidence on MSA in low-income settings in the sub-Saharan Africa region. In addition, the research will provide a deeper understanding of the processes through which MSA is actualized, including the challenges, constraints and enabling factors.

## Purpose

The immediate main goal of ANPPA is to generate evidence on the extent to which and how MSA informs policies related to the implementation of NCD prevention “best buys” in six countries in sub-Saharan Africa: Kenya, Malawi, Nigeria, Cameroon, Togo and South Africa. The evidence generated will catalyze a process of policy engagement by the established network of researchers to support efforts to adopt MSA into policy making and programming.

The specific objectives of ANPPA are (1) To conduct an in-depth assessment in each country about the state of implementation of the NCD “best buys” and of the barriers to their full implementation; (2) To generate robust evidence on the extent to which and how multi-sectoral action is used in the formulation of policies for the implementation of the NCD “best buys” in different contexts, with an emphasis on the population-based interventions; and (4) To build research capacity on multi-sectoral action for health in the region and to set up a networked group of researchers in this area to monitor and assess the effectiveness and impact of multi-sectoral approaches in the long term, and (4) To provide robust evidence to policy and decision-makers in each country to inform the design and implementation of the NCD “best buys”. Ultimately, it is hoped that the evidence generated through ANPPA will be used to promote and adopt multi-sectoral approaches to policy-making for NCD prevention and control in sub-Saharan Africa.

## Conceptual frameworks

We use two frameworks to guide ANPPA: The Walt and Gilson framework of policy analysis [14] and the McQueen analytical framework for inter-sectoral action [15]. The Walt and Gilson framework acknowledges the non-linearity of the policy process as well as the incremental nature of policy making. In this framework, Walt and Gilson focus on four factors: i) The policy contents, ii) the policy actors, iii) the policy processes, and iv) the policy context [14]. The content of particular policies may be examined by looking at the policy objectives, the way the policy is designed, whether there is an accompanying implementation plan and specific mechanisms through which the policy should be actualized. The policy actors will be examined by looking at who was involved in the policy processes, their roles and who else ought to have been involved. The processes include the different stages of the policy making process and the strategies employed to involve different actors. The context may include changes in political climate and management structures; socio-cultural, economic or technological changes; changes in the global financial situation; and conflicting development agendas between governments and development partners.

The framework by McQueen and others is useful in examining the policy processes [15]. The framework focuses on the role of governance in tackling the social determinants of health through the emerging policy practice of “Health in All Policies” that is central to the envisaged NCD prevention efforts. Health-in-all-policies is an approach to public policies across sectors that takes into account the health implications of decisions, seeks synergies, and avoids harmful health impacts in order to improve population health and health equity [16]. The framework by McQueen describes several inter-sectoral governance structures and actions taken by these structures in promoting Health-in-All-Policies. The governance structures include those at central government level (ministerial linkages, cabinet committees and secretariats); parliament level (parliamentary committees); civil service level (interdepartmental committees and units, mega-ministries and mergers); funding arrangements (joint-budgeting, delegated financing) and mechanisms for engagement beyond government with the public, civil society and industry. Depending on the policy, these governance structures may play different roles to initiate or facilitate a policy process and its implementation. These actions include evidence support, setting goals and targets, coordination, advocacy, monitoring and evaluation, policy guidance, financial support, providing legal mandate and actual implementation and management [15].

## Methods

### Study design overview

We will undertake the research using a multiple-case study design as described by Yin [17]. This particular design is suitable since the aim of the research is 1) to provide an in-depth understanding of MSA in multiple African countries and 2) to illuminate a set of NCD prevention-related decisions (that may or may not involve MSA), why they were taken, how they were taken and how they were implemented. The design is appropriate to answer the why and how questions of events and processes over which we, the researchers, have little control. The design will be primarily multiply holistic, and “cases” are conceptualized at multiple levels. At the broadest level, the multiple case study addresses the *global* nature of the policy process across all six countries and all “best buys.” Each country team will conduct its own case study of its own policies; in addition, case studies of each “best buy” (e.g., a case study on tobacco prevention across participating countries) are also included. The primary aim will be replicative logic. For each case we will expect to apply the same methodology to see whether the same outcomes are achieved. In relation to sampling, each case will be selected so that it either predicts similar results (literal replication) or produces contrasting results but for predictable reasons (theoretical replication).

The work will be conducted in two phases. Phase 1 will be the main phase in which all the case study countries will participate. It will include three key activities including: i) a desk review of relevant documents related to the policy formulation process, ii) in-depth interviews with key informants that either participated or should have participated in the policy process, iii) Data analysis of country-level data and a combined analysis of all case studies. More details on these activities are described below.

Phase 2 will be dependent on the findings from phase 1 and the country context. The aim of this phase will be to fill the knowledge gaps in the formulation and implementation of the studied policies and the impact of these policies on population health. Phase 2 will therefore be possible and relevant for countries where: a) The NCD prevention policies studied have been implemented to a significant degree as evidenced by the length of implementation or the milestones already achieved and b) quality data exists to make an assessment of the impact of the policy or policies on population health.

### Formation of research teams

The study is being coordinated by the African Population and Health Research center (APHRC) staff comprising of the Principal Investigator, a research manager, and a research officer. APHRC distributed a call for applications to recruit research fellows to conduct the study in April 2013. APHRC received 17 applications which were reviewed both internally and externally. Of these applications, six were internally reviewed and deemed to be out of scope and hence were not sent for external review. The selection process was based primarily on the strengths and merit of the submitted applications and with consideration to regional and gender representation. APHRC awarded fellowships to 4 senior/mid-level researchers and 5 PhD students identified by the senior researchers from Malawi, Nigeria, Cameroon, and South Africa to conduct case studies on existing NCD prevention policies in their countries. The 5th student is from Togo and is being supervised by the senior researcher from the South African team. Each country’s team includes the research fellow, PhD candidate, a research assistant, and an additional senior researcher to help manage the project in each country. All the country teams’ PhD students, along with their senior researchers, will attend a series of workshops to build their skills and equip them with the right tools to conduct the case studies. In addition to managing the overall the study, APHRC will also conduct a case study on the NCD best buys related policies in Kenya.

### Study implementation

APHRC is providing ongoing guidance to the implementing teams. Quarterly conference with all study teams monitor and guide progress and challenges. To enhance the capacity of the teams to conduct the study, the fellows attended training workshops in Nairobi in October and November of 2013 on policy analysis and case study methodology. In November 2014, a qualitative data analysis workshop was held. There are tentative plans to hold a joint writing workshop for all study teams before the end of 2016.

### Materials

APHRC developed a toolkit to guide the research team in implementing the study. The tool kit includes description of the study background the objectives and the procedures for doing document review, the data collection tool and how to pretest it at country level, ethical considerations, interviewing process, data management procedures and data analysis. This toolkit is available upon request from the corresponding author.

### Data collection

Data collection commenced in the countries from June 2014. Most of the data has been collected and the country teams are now conducting data analysis.

### Document reviews

The aims of the review are to describe the policy context and content, identify existing policies and gaps therein, understand the policy development processes and implementation status. The review covers country-specific policy documents as well as policies from other parts of the world with particular emphasis on identifying those from low and middle income countries and the sub-Saharan Africa region. Document reviews will also include analysis of country-specific quantitative data such as surveys detailing the prevalence of current smokers, proportion of heavy drinkers, or the proportion of adults getting adequate physical activity.

The review will focus on policy documents on NCD prevention (including acts and laws, strategic plans, guidelines and government directives), reviews and case studies of MSA in successful policy formulation and implementation at national level. Relevant policy documents include: ministry website materials such as policy documents, strategic plans, program plans, guidelines, protocols; parliamentary records or debates; local print media for references to policy changes, often as part of speeches by government officials; meeting minutes, activity reports and drafts of policy statements, internal and external memos, meeting agendas and other communications; academic journal articles; relevant donor or non-governmental organization and development partner websites for NCD program reports; libraries and internet search engines. The data to be extracted from documents include identification of years in which relevant policy changes occurred and the events leading up to those decisions. Some key documents may date back to the 1970s or 80s (e.g. national plans and reports).

### Interviews

#### Sampling and recruitment

Participants will be selected using a combination of purposive and snowball sampling [18]. First, sectors and institutions will be identified for inclusion, and then individuals within those sectors/institutions will be identified. Institutions will be purposively selected [18] based on their expected role in NCD policy formulation and implementation. A balance between line government sectors as well as the actors from the state and non-state spheres in each of the sectors will be ensured. Institutions to be included are those that have agreed to participate in the study and provided a letter of permission from the appropriate authority in the sector/department. Individual key research participants to be contacted would include those who participate in the policy making as those who are expected to participate in the process. These individuals will include: Senior decision makers in the selected sectors such as department or division heads or program managers; heads of NGOs or other actors involved in NCD prevention programs or projects; and heads of private sector institutions or departments and programs within those institutions involved in NCD prevention.

Through purposive sampling techniques, key individuals whose names are in the public domain will be identified. Snowball sampling techniques will then be used to identify additional respondents during interviews with the key respondents. After identifying potential participants, invitations will be sent for them to participate in the study through an initial telephone or email contact. If they agree to participate, the information sheet and an outline of the interview will be sent to them in advance of the scheduled interview time.

#### Interviews

Interview guides were developed collaboratively by APHRC and the study teams during the methodology workshop. Interview guides include questions for each policy identified during the document review stage related to the four key “best buys”, including the context in which the policy was developed, the policy content, actors involved in the process, and the implementation status of each policy. In addition, data on how MSA was employed or not, the processes undertaken to ensure MSA, the challenges encountered, what worked and what did not work will be collected. The interview guide was initially piloted in Nigeria and Kenya and revised based on the field experiences. During the training of field workers, each team piloted the guide and the interview guide was revised based on feedback from the pilots. Each country then used the final interview guide with minor adjustments to fit their context if necessary.

The interviews will be conducted at times and venues mutually agreed upon by the research team and the respondents. These venues will be free from distractions and other security risks; and will be conducted in a private place where the conversation cannot be overheard easily by others. All interviews will be conducted in line with the ethical guidelines provided. All interviews will be recorded using a digital recorder. The interviews will last an average of 60 to 90 min.

#### Ethical considerations

All participating scientists have obtained national and institutional approvals through designated and recognized ethical review boards in their countries. All country-specific proposals provided a description of the ethical review process in their countries and institutions as well as explanations on how appropriate safeguards will be put in place to protect the research subjects. Such safeguards included steps to maintain participant privacy and confidentiality and to ensure that interviewees know their participation is voluntary, the risks and benefits of research, and how research findings will be shared. Informed consent will be obtained from the participants prior to conducting the interviews.

### Data management

Qualitative data will be transcribed, cleaned, and saved in word format by the country research teams. A filing system will be set up for each component of the study. Identification codes will be assigned to all individual records including audiotapes, transcripts and demographic information. The data will then be stored on a password-secured hard drive. Copies of the data will be backed up and saved on the existing server for the research project with password protection.

The transcribed interviews will be imported in into the qualitative data management software NVivo. A code book collaboratively created by APHRC and the country teams will guide coding. Other codes that emerge during analysis will also be coded. The coded data from all the countries will then be shared with the research team at APHRC for cross-country analysis.

### Data analysis procedures

We will use content analysis [19] for the qualitative component, guided by the key research questions about the extent and depth of MSA in policy formulation and implementation. The key content areas will be pre-determined based on the policy making framework described above and therefore, the transcribed data will be coded with that in mind. The coding will leave room for other emerging themes outside the established framework. Thematic content analysis will be done manually using the NVivo software. The software will be used to conduct a preliminary analysis to identify text linked with each content area and key themes, which will then be further analyzed in a secondary analysis of data by adding themes or discarding others. Each country’s analysis will also be informed by the analysis from another country that may be ahead in the study process,. In all cases there will be integration of data from different sources.

For the analysis of existing quantitative data, simple descriptive statistics will be used to assess whether there are measurable differences in population level outcomes over time such as prevalence of current smokers, proportion of heavy drinkers, proportion of adults getting adequate physical activity, the proportion consuming adequate amounts of fruits and vegetables, the demand for hypertension treatment. Explanatory statistics such as Mantel Haenszel Chi-Square tests and multivariate regressions will be used to compare differences in proportions and means taking into account contextual factors. More methodological details for phase two will ultimately depend on the availability of data, which will be clearer before Phase 2 begins and which the research fellows will be expected to fully clarify.

### Policy engagement and dissemination

Engagement with policy and decision makers will start early and will take place at all stages of the study from refining the approach in each country, finalizing the tools, collecting the data and sharing the findings. Each country team will convene a policy forum at the inception of the project to share the proposed research and get buy in from potential respondents as well as suggestions on how to improve the research in terms of scope, contextual factors to consider, sources of data, potential key informants as well as agreement on timelines, outputs and outcomes from the research. At the minimum, once the research findings are available there will be a regional working group on MSA for NCD prevention that includes thought leaders, advocates, academics, policy influencers, and professionals from key sectors across the region develop specific actionable policy recommendations on MSA for NCD prevention tailored to different decision makers. Feedback from this working group will be incorporated into a final report; dissemination will then involve active engagement of key stakeholders and policymakers who can directly impact change. Key policy stakeholders will be engaged through international and national level workshops, round table discussions and other public forums that will drive a critical debate on NCD policies. The general public will also be engaged through media coverage and the dissemination of products designed for general consumption. A variety of audience-specific communication products tailored for radio, TV, print media, and online media will be developed to effectively reach the various policy stakeholders as well as build critical public debate around NCD drivers in Africa. In addition we will produce policy briefs, fact sheets, peer reviewed publications, and a research report for sharing with diverse audiences.

## Discussion

We have described the ANPPA study, which aims to generate evidence on the extent to which and how MSA informs policies related to the implementation of NCD prevention “best buys” in six countries in sub-Saharan Africa: Kenya, Malawi, Nigeria, Cameroon, Togo and South Africa. This study builds on the WHO’s recommended ‘whole-of-government’ approach and MSA for development and implementation of national policies and plans for the prevention and control of NCDs [8, 9]. A unique facet of this study is its multiple case study approach that incorporates case studies both across six sub-Saharan African countries and across multiple “best buys” for addressing NCDs (strategies to reduce tobacco use, unhealthy diet, physical inactivity and the harmful use of alcohol).

The ANPPA study will improve our understanding of the WHO’s recommended approach of MSA by specifically seeking to understand the processes national governments use to engage relevant stakeholders in the policy development processes, and to assess the impact of MSA on policy outcome. In addition, the research will provide a deeper understanding of the processes through which MSA is actualized, including the challenges, constraints and enabling factors. We expect that countries that engage in MSA may take longer to enact policies because of the time required to ensure shared understanding among all stakeholders of the problems and the solutions, and that those policies may ultimately be more effective in large part due to that shared understanding. This evidence from different country contexts, and across different “best buys” will inform opportunities for other developing countries to better assess their country’s NCD policies and to implement change effectively and in a way that builds off the experiences of the ANPPA study countries.

The ANPPA study will also contribute to the generation of evidence on MSA in low-income settings specifically in the sub-Saharan Africa region. Given current projections that indicate that by 2020, the largest increase in NCD-related deaths will occur in Africa and by 2030, NCD-related deaths will have exceeded (by 75 %) the combined deaths from communicable diseases, nutritional, maternal and neo-natal deaths [6], this region is particularly appropriate for this study. The process of systematically assessing each country’s NCD-related policies and informing relevant stakeholders to create change will have immediate impact in these countries. Being able to provide lessons learned to other sub-Saharan African countries will also be highly useful.

A few challenges have been identified in the initial implementation of the study. This includes inability to get electronic or hard copies of some of the policy documents, numerous rescheduling of meetings by the key informants, refusals and/or reluctance by some key informants to participate in the study and finally some of the selected key informants required additional approvals from their sector heads before participating in the interviews. By appropriately adjusting to these challenges and limitations, findings from the ANPPA study will not be significantly compromised.

Finally, by illustrating the association between MSA to implement NCD policies and the health status of countries’ citizens, local, regional, and national policy makers, as well as other appointed and elected government officials, non-governmental organizations, and industry stakeholders may find utility to the research findings and conclusions of the ANPPA study. Engagement of policy makers and other stakeholders will ensure future improvements to NCD policies and implementation. Expansion of ANPPA study findings to all interested and influential parties can supplement the potential impact of these findings on a global scale.

## Abbreviations

ANPPA: Analysis of Non-communicable Disease Prevention Policies in Africa
APHRC: Africa Population Health Research Center
MSA: Multi-Sectoral Approach
NCD: Non-communicable Disease
WHO: World Health Organization
